# Light Treatment

**Published:** 1903-12

**Authors:** A. J. Harrison, W. Kenneth Wills

**Affiliations:** Physician to the Skin Department of the Bristol General Hospital


### LIGHT TREATMENT.

BY

A. J. HARRISON, M.B. (Lond.),

*Physician to the Skin Department of the Bristol General Hospital,*

AND

W. KENNETH WILLS, M.A., M.B., B.C. (Cantab.).

---

IN the March number this year (page 23) we published a paper called "Remarks on the 'Light Treatment' of Lupus (Vulgaris and Erythematosus) and Rodent Ulcer, &c.," and at the end of this article we made allusion to some cases of Acne and Paget's disease. We now propose to resume where we left off, and then make some more general remarks about other cases.

There were three cases of acne vulgaris about which details were given. We can say that they were successfully treated with the X-rays, but it is quite clear that fresh infection is not prevented. Thus two of the cases returned after some time in an active condition, but soon improved again. The X-rays do clear up the infected skin, but after-hygienic precautions must be taken to prevent a fresh outbreak. For example, one of the cases became a stoker before a hot furnace, and naturally enough his condition, which had improved till not a comedo or pustule was to be seen on the back, returned to very much the same condition he was in when he first came under X-ray treatment, though now the back is much improving again. The X-rays apparently promote exfoliation and the secretion of the sebum, so that the sebaceous plugs are thrown off with the superficial layers of the epidermis. At the same time, any collection of pus under the skin may be temporarily increased in size. It has been our habit to express as many of the furunculi as possible before each treatment, puncturing the "blind" ones to help their discharge, and then treating the skin antiseptically.

This method proving so satisfactory, a case of "sycosis" was taken in hand. It was a very severe case, abounding in discharging granulomata. Marked improvement quickly followed, but a longer time must elapse before a final result should be reported.

Two cases of intractable eczema, resembling Paget's disease of the nipple, have been much relieved by X-rays. One case involved the right groin and adjoining vulva in a woman about 60. She had always been very healthy, and lived in a very salubrious country city. About two years before she came under our care an irritation commenced in the groin, and gradually spread. Proving very obstinate, it was cauterized (thermo-electric) on two occasions by her medical man, but the irritation returning, she was sent under our care. Under the X-rays it quickly healed over, and the intense itching diminished, but small spots would break down again, especially where the cauterization had been; but ultimately the whole healed apparently soundly, and now nearly a year has elapsed and we have had the information that the improvement remains quite permanent.

The other case is that of a man aged 54, in whom the right angle of the mouth and the adjacent mucous membrane were involved. He was sent to Dr. Harrison by a medical man in Bath in March, 1899, under the impression that the condition might be malignant. This it clearly was not, and under treatment it improved, but very readily broke down again. Looking back now at the early stages of Paget's disease, such condition seems to explain the recurring raw, scaly, fissured appearances. In 1902 he was healed with the "light treatment," and later on with X-rays, and soon great improvement manifested itself, and treatment ceased for a time; but he has had two or three slight relapses, which were readily relieved, and as it is now some months since he presented himself, it is reasonable to conclude that he is keeping all right. *Apropos* of these cases, we have had a case in private of Paget's disease in the right nipple region in a man. It was of several years' duration, but under the X-rays it improved very rapidly, and appears to be nearly cured.

A case of eczema barbæ is worth associating with the above cases. It is still under treatment, but the way in which the disease cleared off on the right side of the face with the exfoliation of the hairs, after the production of a slight erythematous reaction, was very remarkable. The skin was, and is still, absolutely smooth and clean.

There have been 50 cases of lupus vulgaris in the department since December, 1901, and with these should be included five cases in which the diagnosis was not certain.

There is not one of these cases of genuine Lupus vulgaris which has not received some, if not considerable, benefit, but it should be admitted at once that in a few cases no very great diminution in the area affected has taken place. This may probably be accounted for, first, by the length of time the disease has been present (48 years in one case); secondly, by the depth to which the individual nodules have extended; and, thirdly, many of the cases have been subjected to more or less severe surgical procedures, which render the penetration of the light much more difficult, as the scar tissue formed over the disease deposit is like so much ground glass in the front of a light, diffusing the rays and thereby making them less effective. Yet in these cases improvement has taken place, in the sense that little, if any, extension has occurred during the treatment, ulcerations have healed, and, what is of great comfort to the patients themselves, the cold, dry winds, which crack and fissure the affected parts to a terrible degree, have scarcely had any deleterious effects. No doubt curative results are much influenced by the kind of infection with which we have to deal. A diffuse staining of the skin, with no definite nodules or isolated deposits, is the most unyielding to light or the X-rays. This form of the disease is not so common as the nodular, and has wrongly been diagnosed as Lupus erythematosus, whereas it is an erythematous form of Lupus vulgaris. It is surrounded by a slightly raised margin, and displays scales almost seborrhœic in character. The skin is easily affected by the light reactions, blistering extensively, yet when the reaction has passed off scar tissue is by no means always left behind. In these cases Dr. Hall-Edward's treatment of painting the cleansed parts with an aqueous solution of potassium permanganate gives satisfaction, in that it removes the scales and leaves the surface smooth, and in some cases supple. It is our practice to continue the "light treatment" so long as there is disease in the skin, and to treat the mucous membranes with the X-rays; but where the skin is ulcerated or extremely infiltrated, or where reactions are hard to obtain, periodic exposures are given with the X-ray tube in addition; and it is found that under this method ulcerations quickly heal, thickened indurated parts are reduced to normal size. and reactions are much more easily obtained. Cases of L. hypertrophicus—of which terribly disfiguring disease several have been treated in the department—well exemplify this statement. The soft, almost villous, granulations which characterise the disease retrogress and leave white scars behind, scars cleaner and smoother when no surgical scraping has been previously practised. When improvement like this is attained it is often very desirable to send the patients into the country when practicable, and give them a month's rest. In this way the reaction erythema and any blistering die down, making it easier to see any remaining foci of disease. Although this adds time to the treatment, yet there is very distinct gain in the end.

We have classified the cases in the following way :—

**Lupus Vulgaris.**—50 cases, and one uncertain in diagnosis.

| | | | |
|---|---|---|---|
| I. | Relieved ... ... ... ... ... ... ... ... ... ... ... | | 7 |
| II. | Condition on skin relieved, but disease persisting elsewhere : | | |
| | (*a*) Ulceration inside nose ... ... ... ... ... | 4 | 6 |
| | (*b*) Dactylitis, under treatment... ... ... ... | 1 | |
| | (*c*) ,, and lachrymal sac abscess (tubercular) ... ... ... ... ... ... | 1 | |
| III. | Discharged, improved very greatly, though probably not quite cured ... ... ... ... ... ... ... ... ... | | 2 |
| IV. | Improved, but still under treatment, some nearly well | | 22 |
| V. | Ceased treatment, improved, some very slightly .. | | 9 |
| VI. | Ceased treatment *pro tem.*, though improved (illness, &c.)... ... ... ... ... ... ... ... ... ... ... ... | | 2 |
| VII. | Unsatisfactory through non-attendance ... ... ... | | 2 |
| VIII. | Unimproved ... ... ... .. ... ... ... ... ... ... | | 1 |
| IX. | "Recurrence" after apparently relieved ... ... ... | | 3 |
| X. | Too soon to report anything... ... ... ... ... ... ... | | 1 |

In this table four cases are included under two headings.

It will be observed that V. contains nine cases. In one case the diagnosis was uncertain. It improved under the X-rays, but not under the "light treatment," and seemed more like a rodent ulcer: the disease was situated in the upper lip and septum of the nose.

We have treated several cases of tuberculous ulceration and dactylitis with the X-rays with distinct improvement. The discharge lessens, and the sinuses tend to heal. In one case healing occurred under the X-rays where there were ulceration and sinuses in the groin; and though the lad passed through a slight attack of diphtheria last April, the parts have not broken down.

Two cases of a warty, discharging condition of the thumb, very much resembling verruca necrogenica, have been treated with X-rays, and with excellent results: one is nearly well, and the other has shown no return since last seen on August 1st.

**Lupus Erythematosus.**—In all six cases have been treated. One case, very incipient, was relieved entirely; another left for treatment elsewhere; and the rest show only very slight improvement. One of them has improved by directing the light at a distance, and without any pressure, upon the diseased area, a method suggested by Norman Walker.

**Rodent Ulcer.**—Eighteen cases. I.—Relieved entirely, but still kept under observation, 8. II.—Improving under treatment, 7. III.—Unsatisfactory through non- or bad attendance, 2. IV.—Probably made worse, 1. This last may have been carcinoma; if rodent, and the sections made of small tags of growth were not diagnostic, it was of very rapid growth both before and during treatment. The subject was an old man of 78, very decrepit, and the disease is said to have occurred after an accident. Two months before he came to the hospital it started as a hard lump on the left ala of the nose, and very soon it began to fissure and discharge. In spite of treatment, the disease advanced rapidly until the infra-orbital notch was reached. Then the nerve became affected, and the pain was intense. The X-rays relieved the neuralgia for a short time, and this relief was the chief reason for their continuance; but the disease still advanced.

Another case (H. L., 73) was one noted in the March paper, in which there was extensive destruction of the superior maxilla, nose, and cheeks. His condition steadily improved, but as granulations began to appear round the upper central incisors these were removed, and revealed disease at the back of the tooth roots. The "rodent" condition seems relieved, but time must elapse to prove this. Bands of adhesion, especially on the left side, prevent the opening of his mouth wide enough to admit of thorough digital examination. He is being kept under observation, and is in excellent condition so far as his day's work is concerned.

Unless erythematous reactions occur in the treatment of rodent ulcer, very little good is done: they need not be severe, but the more pronounced they are up to a certain extent the more rapid is the healing. But eventually this reaction wears itself out, and another may be produced; if not—and this is mostly to be noticed in aged patients—the ulcer is apt to persist in spite of treatment. A good example of this has occurred in an old and very frail woman of 73. She came under observation in February, 1902, and rapidly improved under treatment at first. The ulcer—about the size of half a crown, on the right cheek and lip, with hard, warty, everted edges—had commenced about 20 years previously, and had never had surgical treatment. It filled up and healed fairly speedily, but three nodules remained. These have been very difficult to remove, doubtless greatly increased by the frail condition of the patient, and by her consequent inability to attend the hospital regularly; and when she did she could not stand any protracted exposure. There is still remaining one small nodule and one spot of ulceration. The hard, solid edges are found to disappear, but go the sooner if they are touched with pure carbolic acid or are lightly scarified.

Cases of favus and tinea tonsurans have been treated by the X-rays for depilatory purposes with good results. Another interesting case was that of a woman who had carcinoma of the cervix and vagina, with fistula and incontinence of urine. She developed very extensive and acute septic (?) pemphigus. Acting upon the suggestion of Max Heine, the blebs were rayed at a distance, by means of the London Hospital lamp. The blebs dried up and irritation ceased after $\frac{1}{4}$-hour exposure. Some fresh ones came out later, but the skin condition was certainly improved. Where the urine irritated the skin fresh crops of blebs were constantly coming out in spite of the "light treatment." The patient died shortly after from the carcinoma.

The pains occasioned by carcinoma are undoubtedly much relieved by ray treatment This relief will last for from 12 to 24 hours, and can be obtained again and again.

---

